# Impact of adoption of safety devices in reducing accidents at work with biological material

**DOI:** 10.1186/1753-6561-5-S6-P223

**Published:** 2011-06-29

**Authors:** CT Zogheib, MM Baraldi, MM Simonetti, CM Santoro

**Affiliations:** 1 Serviço de Controle de Infecção Hospitalar, Hospital Alemão Oswaldo Cruz, São Paulo, Brazil

## Introduction / objectives

Injuries from needles and sharps by health professionals, are considered extremely dangerous because they are potentially capable of transmitting microorganisms, and the human immunodeficiency virus (HIV), Hepatitis B and Hepatitis C, are the infectious agents most commonly involved. Institutions should seek to minimize risk as much as possible through measures including the installation of safety devices.

## Methods

Observational study in a general hospital, analyzing workplace accidents with biological material in the period 2003 to 2010, before the sharps injuries related to manipulation of the venous needle, performing blood glucose testing with a lancet and capillary puncture vein without safety devices and analyzes the situations mentioned before and after the implementation of safety devices. The initiative of the Infection Control Comission in deploying these devices was to participate in the process, rigorous evaluation of these materials and the training of all health care team.

## Results

Of the 398 accidents with biological materials and accompanied with the implementation of security devices including: the infusion tube systems had 90% reduction in accidents involving needles, lancets with the adoption of a security system for performing blood glucose monitoring, had 100% reduction in accidents, and venipuncture devices safely, we had 97% reduction in accidents.

## Conclusion

Initiatives to make the everyday activity of professionals safer, preventing the risk of occupational accidents with biological material can minimize the possibility of acquisition of work-related diseases, are important actions taken by healthcare institutions.

## Disclosure of interest

None declared.

